# Microbial Reduction of Chromate in the Presence of Nitrate by Three Nitrate Respiring Organisms

**DOI:** 10.3389/fmicb.2012.00416

**Published:** 2012-12-17

**Authors:** Peter Chovanec, Courtney Sparacino-Watkins, Ning Zhang, Partha Basu, John F. Stolz

**Affiliations:** ^1^Department of Biological Sciences, Duquesne UniversityPittsburgh, PA, USA; ^2^Department of Chemistry and Biochemistry, Duquesne UniversityPittsburgh, PA, USA; ^3^Center for Environmental Research and Education, Duquesne UniversityPittsburgh, PA, USA

**Keywords:** chromate reduction, nitrate respiration, bioremediation, nitrate reductase, nitrite reductase

## Abstract

A major challenge for the bioremediation of toxic metals is the co-occurrence of nitrate, as it can inhibit metal transformation. *Geobacter metallireducens*, *Desulfovibrio desulfuricans*, and *Sulfurospirillum barnesii* are three soil bacteria that can reduce chromate [Cr(VI)] and nitrate, and may be beneficial for developing bioremediation strategies. All three organisms respire through dissimilatory nitrate reduction to ammonia (DNRA), employing different nitrate reductases but similar nitrite reductase (Nrf). *G. metallireducens* reduces nitrate to nitrite via the membrane bound nitrate reductase (Nar), while *S. barnesii* and *D. desulfuricans* strain 27774 have slightly different forms of periplasmic nitrate reductase (Nap). We investigated the effect of DNRA growth in the presence of Cr(VI) in these three organisms and the ability of each to reduce Cr(VI) to Cr(III), and found that each organisms responded differently. Growth of *G. metallireducens* on nitrate was completely inhibited by Cr(VI). Cultures of *D. desulfuricans* on nitrate media was initially delayed (48 h) in the presence of Cr(VI), but ultimately reached comparable cell yields to the non-treated control. This prolonged lag phase accompanied the transformation of Cr(VI) to Cr(III). Viable *G. metallireducens* cells could reduce Cr(VI), whereas Cr(VI) reduction by *D. desulfuricans* during growth, was mediated by a filterable and heat stable extracellular metabolite. *S. barnesii* growth on nitrate was not affected by Cr(VI), and Cr(VI) was reduced to Cr(III). However, Cr(VI) reduction activity in *S. barnesii*, was detected in both the cell free spent medium and cells, indicating both extracellular and cell associated mechanisms. Taken together, these results have demonstrated that Cr(VI) affects DNRA in the three organisms differently, and that each have a unique mechanism for Cr(VI) reduction.

## Introduction

Chromium (atomic number 24) is an enigmatic transition metal that is abundant (average concentration ∼100 μg/g) within the Earth’s crust, yet has no known natural biological role. Chromium is found in silicates as well as in mixed metal minerals, and is enriched in certain soils (Cox, 1995). Like the other group VI metal ions, such as molybdenum and tungsten, chromium can exist in a variety of oxidation states, with Cr(III) and Cr(VI) as the most prevalent. Trivalent chromium, Cr(III), occurs naturally in rocks, plants, and soil. Hexavalent chromium, Cr(VI), is a common by-product of industrial processes, e.g., electroplating, leather tanning, and wood preservation, and in nuclear waste (Pattanapipitpaisal et al., 2001). Unfortunately, Cr(VI) is also the most mobile and the more toxic form of chromium. The mobility of Cr(VI) in the environment is due to the intrinsically high water-solubility. Cr(III) is insoluble at physiological pH and thus less mobile (Ball and Nordstrom, 1998). The mechanism of chromium toxicity in humans is not completely understood, however, Cr(VI) is believed to be more toxic than Cr(III) due to its ability to get into the cells using phosphate transporters. Cr(VI) is a well-known mutagen and potential carcinogen (Losi et al., 1994). Because Cr(III) exhibits less toxicity and reduced mobility within the environment, many bioremediation strategies focus on the transformation of Cr(VI) into Cr(III) (Kanmani et al., 2012).

The chemical transformation of Cr(VI) into Cr(III) is well understood. From a chemical view point, Cr(VI) is more stable under oxidizing conditions and Cr(III) is more stable under reducing conditions. Chromium speciation is also dependent on pH with Cr(III) more prevalent at neutral pH, although multiple species of chromium do exist in equilibrium (Ball and Nordstrom, 1998). Altering the redox environment of chromium contaminated sediments, to stabilize Cr(III), is a potential means for Cr(VI) remediation. The challenge of redox induced Cr(III) precipitation is creating a practical method for treating large areas of contaminated sediment and water, without significantly impacting the natural biota.

Many remediation strategies are based on microbial transformation (Turick et al., 1996; Viamajala et al., 2002a,b; Camargo et al., 2003a,b; Kanmani et al., 2012) and have included schemes involving microbial consortia (Pattanapipitpaisal et al., 2001; Battaglia-Brunet et al., 2002; Arias and Tebo, 2003; Cheung and Gu, 2003), and pure cultures (Nepple et al., 2000; Park et al., 2000; McLean and Beveridge, 2001; Wielinga et al., 2001; Kwak et al., 2003). The microbial reduction of Cr(VI) to Cr(III) has been demonstrated in both facultative aerobes, e.g., *Escherichia coli*, *Shewanella alga, Bacillus* sp., *Ochrobactrum* *tritici, Pseudomonas putida, Cellulomonas* sp., and strict anaerobes, e.g., *Geobacter metallireducens*, *Desulfovibrio vulgaris*. These bacteria appear to utilize different molecular mechanisms for Cr(VI) reduction (Lovley et al., 1993; Park et al., 2000; Branco et al., 2008). Aerobic Cr(VI) reduction is mediated by several different soluble cytoplasmic enzymes. Suzuki et al. (1992) described the purification of a NAD(P)H-dependent Cr(VI) reductase enzyme from *Pseudomonas ambigua*; which was later shown to be a homolog of nitroreductase (Kwak et al., 2003). A smaller (20 kDa) enzyme purified from *P. putida* was also found to be NAD(P)H and NADH dependent and inhibited by sulfate (Park et al., 2000). Anaerobic reduction of Cr(VI) can also be mediated by several different redox active proteins including low potential c-type cytochromes and hydrogenases (Michel et al., 2001; Chardin et al., 2003). Studies on *Desulfovibrio* species identified a tetraheme c-type cytochrome with Cr(VI) reduction activity (Chardin et al., 2003). Other heme-c proteins that have been implicated in Cr(VI) reduction include an octaheme cytochrome c_3_ (Czjzek et al., 1996), a multiheme (16) cytochrome Hmc (Bruschi et al., 1992), and a non-heme cytochrome (Saraiva et al., 1999). The sulfate-reducing *Desulfovibrionaceae* family are also known to utilize hydrogenases in Cr(VI) reduction (Lovley and Phillips, 1994; Chardin et al., 2003).

Currently, the implementation of microbial chromium bioremediation has encountered two challenges: (1) the sensitivity of the microbe to elevated levels of chromium and (2) the inhibitory effects of co-contaminates, such as nitrate, on the reduction of Cr(VI) to Cr(III). Several chromium resistant microbes have been identified (Morales et al., 2007; Mehta and Vaidya, 2010). In *Rhodobacter sphaeroides*, chromium resistance has been linked to reduction via a non-specific FADH_2_-dependent metal reductase, and is thought to be for internal redox control and detoxification (Moore and Kaplan, 1992, 1994). It is important to note that Cr(VI) resistance is not necessarily coupled to Cr(VI) reduction, but rather to an efflux system to remove chromium from cells (Nies and Silver, 1995). Thus an organism’s response to Cr(VI) can be different (Ramírez-Díaz et al., 2008). Further complicating the situation is the fact that many contaminated sites not only contain chromium, but also elevated levels of other highly oxidized chemicals, such as nitrate. The biotransformation of chromium in complex mixtures can be impacted in three ways: (1) competitive alternative electron acceptor (inhibiting transformation), (2) co-metabolism (i.e., concomitant reduction, stimulating transformation), and (3) induction of specific proteins and pathways involved in oxidation/reduction reactions (stimulating transformation). The third scenario would be the most desirable for bioremediation of nitrate and chromate contaminated environments.

We investigated the effect of Cr(VI) reduction on dissimilatory nitrate reduction to ammonia (DNRA) by soil bacteria because nitrate is commonly found with Cr(VI) at many contaminated sites. Bacteria reduce nitrate into nitrite with nitrate reductase enzymes (Nap or Nar), then nitrite can be transformed into ammonia during DNRA or to nitric oxide during denitrification, by nitrite reductase enzymes (Stolz and Basu, 2002). Nitric oxide may be further enzymatically reduced to nitrous oxide and dinitrogen gas in the latter process (Zumft, 1997). These two different dissimilatory processes, DNRA and denitrification, yield different amounts of energy and reducing equivalents. Nevertheless, nitrate is a powerful antagonist of many metal and metalloid transformations, as it may compete for the available electron donor or act directly by interfering with the enzymatic process. Cr(VI) is energetically less favorable when compared with nitrate (Ball and Nordstrom, 1998), thus nitrate should inhibit respiratory chromate reduction. Herein we report the impact of nitrate reduction on Cr(VI) transformation. To investigate these processes, we have focused on three dissimilatory metal reducing organisms that are capable of DNRA: *G. metallireducens* (Lovley et al., 1993), *Desulfovibrio desulfuricans* strain 27774 (Liu and Peck, 1981), and *Sulfurospirillum barnesii* (Oremland et al., 1994).

Despite their shared ability to carry out DNRA, the organisms use different nitrate reductases. *G. metallireducens* reduces nitrate to nitrite via the membrane bound nitrate reductase (Nar), while *D. desulfuricans* strain 27774 and *S. barnesii* have periplasmic nitrate reductases (Nap); their catalytic subunit (NapA) differs significantly in size with *D. desulfuricans* the smallest at ∼70 kD (Marietou et al., 2005) and *S. barnesii* the largest at ∼102 kD (Sparacino et al., 2008; Sparacino-Watkins, 2011). All three organisms have a pentaheme nitrite reductase (Nrf) that reduces nitrite to ammonia. We demonstrate here that dissimilatory nitrate reduction in each of these organisms is affected differently by the presence of Cr(VI) and involves different mechanisms for attenuation.

## Materials and Methods

### Cultures and growth experiments

*G. metallireducens* was grown on freshwater acetate medium with nitrate (FWA-NO_3_) as previously described (Lovley et al., 1993) and modified in Senko and Stolz (2001). *S. barnesii* and *D. desulfuricans* were grown on SES3 freshwater medium with lactate and nitrate as previously described (Stolz et al., 1997) or FWL-NO_3_, a modified FWA-NO_3_ medium in which lactate (10 mM) was used as a replacement for acetate as the electron donor and carbon source.

FWA-NO_3_ and FWL-NO_3_ (for growth of *G. metallireducens* and *D. desulfuricans* respectively) contained 1.5 g NH_4_Cl, 0.6 g NaH_2_PO_4_, 0.1 g KCl, and 2.5 g NaHCO_3_, 2 mL of 500× trace element solution and 2 mL 500× vitamin mix (Lovley and Phillips, 1988) in ∼1 L (996 mL) of distilled water. The medium was adjusted to pH 6.8 with HCl and dispensed into Wheaton bottles (either 50 or 125 mL), and degassed with 80:20 N_2_:CO_2_ (5 min for the liquid, 2 min for the headspace). Bottles were sealed with butyl rubber stoppers and capped with aluminum crimp tops before autoclaving. FWA-NO_3_ contained acetate (6.8 g/L) and NaNO_3_ (1.7 g/L). FWL-NO_3_ was amended with lactate (2.72 mL of a 60% stock solution) and NaNO_3_ (1.7 g/L). The SES3 medium contained 0.23 g K_2_HPO_4_, 0.23 g KH_2_PO_4_, 0.46 g NaCl, 0.23 g (NH_4_)_2_SO_4_, 0.12 g MgSO_4_.7H_2_O, 10 g yeast extract, and 4.2 g NaHCO_3_, lactate (2.72 mL of a 60% solution), nitrate (as sodium nitrate 1.7 g) and 2 mL 500× trace elements, 2 mL 500× vitamin mixture (as above) in ∼1 L (996 mL) of distilled water. The medium was adjusted to pH 7.2 with HCl and dispensed into serum bottles, either 50 or 125 mL (Wheaton), and degassed with 80:20 N_2_:CO_2_ (5 min for the liquid, 2 min for the headspace). Bottles were sealed with butyl rubber stoppers and capped with aluminum crimp tops before autoclaving.

The vitamin mixture (500×; Lovley and Phillips, 1988) contains biotin, 10 mg/L; folic acid, 10 mg/L; pyridoxine HCl, 50 mg/L; riboflavin, 25 mg/L; thiamine 25 mg/L; nicotinic acid, 25 mg/L; pantothenic acid, 25 mg/L; *p*-aminobenzoic acid 25 mg/L; thioctoic acid, 25 mg/L; B12, 0.5 mg/L. The mineral mixture (500×) contains nitrilotriacetic acid, 7.5 g/L; MgSO_4_, 15.0 g/L; MnCl_2_, 2.22 g/L; NaCl, 5.0 g/L; FeCl_3_, 0.335 g/L; CaCl_2_.2H_2_O, 0.5 g/L; CoCl_2_, 0.5 g/L; ZnSO_4_.7H_2_O, 1.36 g/L; CuSO_4_.5H_2_O 0.05 g/L; AlK(SO_4_)_2_, 0.05 g/L; granular boric acid, 0.05g/L; Na_2_MoO_4_, 0.125 g/L; NiCl_2_.6H_2_O, 0.125 g/L; Na_2_WO_4_, 0.125 g/L.

Stock solutions of potassium chromate were prepared separately, degassed to remove oxygen, filter sterilized, and then added aseptically, using the Hungate technique, to autoclaved media to a final concentration of 100 μM Cr(VI). The growth kinetics were determined by turbidity (OD at 600 nm) on a Perkin Elmer Lambda 2 dual beam spectrophotometer. Kinetic data for growth and chromium reduction were analyzed by Origin 7.5 using non-linear functions using Levenberg–Marquardt algorithm and simplex method implemented in Origin 7.5. Each data set was fit with a single exponential function (*y* = *A*_1_ × exp(−*x*/*t*_1_) + *y*_0_ for decay or *y* = *A*_1_ × exp(*x*/*t*_1_) + *y*_0_ for growth) indicating a first order process. Some data pertaining to the Cr reduction as a function of time were also analyzed by Graph Pad Prism software.

### Live/dead whole cell and filtrate assays

Cells of *G. metallireducens*, *D. desulfuricans*, and *S. barnesii* were grown on nitrate as described above. Three milliliters samples of cells, in log phase of growth and comparable cell numbers, were harvested by centrifugation, washed in Tris buffer (10 mM, pH 7.4), then resuspended in 3 mL of fresh medium. Samples (3 mL) of supernatant were collected via degassed syringe and filtered using 0.2 μm polycarbonate Millipore filters and stored under nitrogen gas. The cell suspensions or filtrates (3 mL each) were then added to 50 mL of fresh medium amended with 100 μM Cr(VI) and incubated for 48 h. Heat killed controls were autoclaved for 20 min. Aliquots were removed at 0, 2, 6, 12, 24, and 48 h, and Cr(VI) concentration determined by the diphenylcarbazide (DPC) method as previously described (Saltzman, 1952).

### Electron donors for chromium reduction in *D. desulfuricans*

Cells of *D. desulfuricans*, grown of FWL-NO_3_ medium, were harvested by centrifugation, as previously described, then resuspended in 180 mL bicarbonate buffer at pH 6.8. Serum bottles (50 mL) were grouped in triplicate as: control (no cells and no electron donor); cells only, cells with lactate, cells with hydrogen, lactate only, and hydrogen only. The cell suspension (20 mL) was then added to each bottle in the three groups – cells only, cells with lactate, cells with hydrogen. Bicarbonate buffer (20 mL) was added to each bottle in the following groups: positive control, lactate only, and hydrogen only. All bottles with the exception those with hydrogen were degassed with N_2_, while hydrogen bottles were degassed and had their headspace filled with H_2_. In the lactate samples, 200 μL of 1 M lactate was added. Each bottle was sterilized by autoclaving, and was crimp sealed to maintain anaerobic condition, then 200 μL of the 10 mM Cr(VI) stock solution was added to initiate the reduction. At each time point, 3 mL of sample was withdrawn and the Cr(VI) content was determined by DPC assay. From each withdrawal three measurements were conducted providing three technical replicates for each biological replicate.

### Chromium resistance in *D. desulfuricans*

*D. desulfurican* was grown for 48 h, then 20 mL of the culture was transferred to five 25-mL serum bottle under anaerobic conditions. Different concentrations of potassium chromate were then added to each bottle to a final concentration of 25, 50, 75, 100 μM. In the control experiment, FWA-NO_3_ medium was used with potassium chromate without any cells. Cell density was monitored at 600 nm absorbance with Perkin Elmer UV/VIS spectrometer. Aliquots were withdrawn at a regular time intervals and Cr(VI) was determined by the DPC assay.

### Activity and chemical assays

Protein concentration was measured using the DC protein assay kit (Bio-Rad). Bovine serum albumin (Pierce) was used as a standard. All enzyme activity assays (nitrate, nitrite, and chromate reduction) were performed using the viologen-coupled reductase method (Stolz et al., 1997) with methyl or benzyl viologen. In-gel activity assays were performed using native gel electrophoresis to first separate proteins, prior to methyl viologen coupled activity assay analysis (Afkar et al., 2003). Protein electrophoresis was performed under non-denaturing conditions (the polyacrylamide gel contained 0.5% of CHAPS, the sample buffer contained 0.7% CHAPS and anode buffer 0.1% CHAPS) according to standard protocols. The in-gel assay was performed in 50 mM Tris-Cl (pH 7.9), with 10 mM methyl viologen as the electron donor, and KNO_3_ [0.1 M], KNO_2_ [10 mM], or K_2_CrO_4_ [0.7 mM] as the electron acceptors in presence of sodium dithionite. Images of the activity gels were documented using a densitometer (GS-800, Bio-Rad). After the activity assay was finished, gels were incubated for 15 min in 12.5% trichloroacetic acid (TCA) before staining with colloidal Coomassie stain.

Microscale solution assays were performed inside an inert atmosphere glove box (Vacuum Atmospheres) at 22°C using the ELx808 absorbance microplate reader (BioTek). The oxidation of methylviologen as a function of time was monitored at 630 nm and the data were analyzed using Gen5 Data Analysis Software (BioTek). The final reaction contained 3 mM methyl viologen or benzyl viologen in 100 mM Tris-HCl buffer at pH 7.6. A 60-mM sodium dithionite stock solution was used to reduce the viologen reaction solution by titrating to a final absorbance of 1.2–1.6 at 630 nm. All assay solutions were purged with nitrogen or argon gas for 20 min, and then sealed before transfer into the anaerobic chamber. The enzyme was incubated with the reduced benzyl viologen for 10 min and the absorbance was monitored to ensure the rate of oxidation was insignificant prior to addition of substrate. The reaction was initiated by the addition of substrate (nitrate, chromate, or nitrite) and the absorbance was monitored until the reaction stopped. Under the given conditions, the rate of reduced benzyl viologen oxidation by the protein sample alone (i.e., no substrate) was found to be negligible. Activities were calculated as μmoles of viologen oxidized per min using the extinction coefficient of 13 mM^−1^cm^−1^ and 7.4 mM^−1^cm^−1^ for methyl- and benzyl-viologen, respectively (Jones and Garland, 1977). The rate of viologen oxidation was determined by plotting the concentration of reduced viologen over time (seconds). Linear regression (GraphPad Prism) of the initial data points (first 90 s after adding substrates) was used to calculate the rate of viologen oxidation.

Nitrite concentration was determined using the microtiter plate-based Griess assay (Borcherding et al., 2000). KNO_3_ (Fluka) was used as a standard for nitrite estimation, within the range of 1.6–100 μM. Cr(VI) concentrations were determined using the DPC method (Saltzman, 1952). The standard reaction mixture contained 940 μL sample, 50 μL 3 M H_2_SO_4_, and 10 μL 2.5% DPC in acetone.

For selected samples, media were amended with 100 μM Cr(VI) and aliquots (60 μL) were collected in 0, 24, 48, 72, 96, and 120 h. The samples were spiked with ^50^Cr(III) and ^53^Cr(VI) isotopes using isotope dilution mass spectrometry (MS) with EPA method 6800 on a Shimadzu ICP-MS (Kingston et al., 1998, 2005). Cr(VI) and Cr(III) concentrations were determined using Chromium Speciated Analysis Calculation Software (Applied Isotope Technologies, Inc.).

### Proteins purification and identification

Preparative CHAPS native-polyacrylamide gel electrophoresis (CN-PAGE) was used to separate proteins involved in nitrate reduction and Cr(VI). CN-PAGE was used to purify the multiheme cytochrome c from nitrate grown cultures of *G. metallireducens*. A solid cylindrical (tube) gel, 2.5″ diameter, was created by casting the gel inside a modified Model 491 PrepCell (Bio-Rad) as reported (Martínez Murillo et al., 1999). Briefly, the resolving and stacking gels contained 0.5% of CHAPS, the sample buffer contained 0.7% CHAPS, the anode buffer 0.1% CHAPS, and the cathode buffer contained no CHAPS. Electrophoresis was performed using cold buffer and separation was performed using 100 V for at least 3 h. The entire tube gel was removed, washed with DI water, and then separated into small disk shaped sections, which were diced into ∼1 cm cubes prior to electroelution. Electroelution was conducted using the Elutrap™ System (Whatman) according to the manufacturer’s guidelines. The diced gel samples were immersed in the CHAPS anode buffer. Electrophoresis was conducted at 100 V for 1 h. SDS-PAGE was used to determine the relative molecular mass of the proteins, with a 4% acrylamide stacking gel and a 12% acrylamide resolving gel on a Mini-Protein gel system (Bio-Rad, Hercules, CA, USA). After colloidal coomassie blue staining, the gel was scanned with a Bio-Rad GS-800 densitometer and the molecular masses of the subunits calculated relative to the molecular weight standards (Quantity One, Bio-Rad). The electronic spectra for air-oxidized, dithionite reduced (13 mM Na_2_S_2_O_4_), and chromate oxidized (3 mM K_2_CrO_4_) cytochrome was determined on a Cary 3 spectrophotometer.

Cells of *S. barnesii* were grown on 20 mM nitrate and 15 mM lactate medium in a New Brunswick Scientific Microferm anaerobic fermentor (14 L). Cells (9–13 g total) were harvested in the log phase of growth (∼12 h) and lysed by French Pressure Cell. Protease inhibitor cocktail (Sigma Chemical, St. Louis, MO, USA) and DNAse were added to cold lysate immediately after lysis. The membrane fraction was obtained by ultracentrifugation (100,000 *g*). The membrane pellet was suspended in 50 mM Tris-Cl buffer pH 7.7, and then treated with detergent, either 3-[(3-Cholamidopropyl)dimethylammonio]-1-propanesulfonate (CHAPS) or *n*-octyl-β-d-glucopyranoside (OBGP) to solubilize membrane proteins. Insoluble proteins were removed by ultracentrifugation (100,000 *g*). The CHAPS soluble supernatant was subjected to subsequent ammonium sulfate precipitation (30% saturation), and the supernatant was collected again via centrifugation (50,000 *g* Beckman Coulter Ultracentrifuge) and then dialyzed to lower the salt concentration. The desalted fraction was loaded onto a DEAE anion exchange column and eluted with a NaCl gradient of 300 mM NaCl in 300 mM Tris-HCl. A non-specific metalloid reductase, Rar, was solubilized from the membrane suspension with OBGP and enriched by subsequent ammonium sulfate precipitation. In-gel activity assays were done using CN-PAGE with the Mini-Protein gel system as described above.

Protein identification was made using in-gel trypsin digestion and Matrix Assisted Laser Desorption Time of Flight (MALDI-TOF) MS. Sample preparation for MALDI-TOF MS analysis was carried out using the methods described in Shevchenko et al. (2006). α-Cyano-4-hydroxycinnamic acid (Fluka, Switzerland) was used to prepare the matrix solution at a concentration of 10 mg/mL in 70% acetonitrile/0.1% trifluoroacetic acid. The peptide solution (0.5 μL) was deposited onto the MALDI target followed by 0.5 μL of matrix solution. The MALDI-TOF MS was performed on a Voyager-DE STR MALDI-TOF mass spectrometer (Applied Biosystems, Foster City, CA, USA) in the reflector mode at the Genomics and Proteomics Core Lab of the University of Pittsburgh. External mass calibration was achieved utilizing calibration mixture 2 of the Sequazyme peptide mass standard kit (Applied Biosystems, Foster City, CA, USA). Proteins were identified from the NCBInr database by Mascot (http://www.matrixscience.com). Protein identities were assigned if the protein score was significant with stringency of 0.6 Da. Search parameters included carbamidomethylation of cysteine, possible oxidation of methionine, and one missed cleavage site.

## Results

### The effect of chromate on bacterial growth

**Figure 1:**
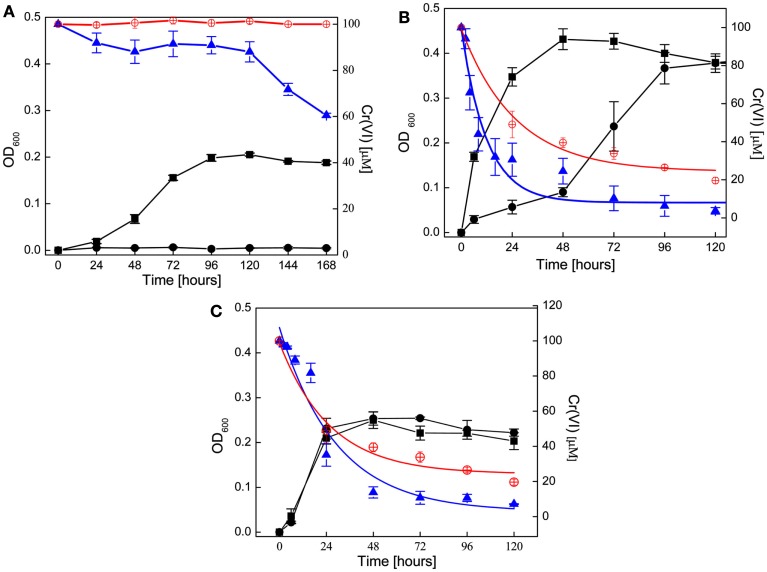
**Cell growth on nitrate in the presence of chromate (●) as compared to nitrate alone (■)**. *G. metallireducens* **(A)** was grown on FWA-NO_3_ while *D. desulfuricans* **(B)**, and *S. barnesii* **(C)** were grown on SES3 medium. Decrease in chromate concentration in living cultures is shown as (▲). Chromium concentration in control experiments under abiotic conditions **(A)** [FWA-NO_3_ medium **(B,C)**, in SES3 medium without inoculum] is shown as (⊕). The rate of change in Cr(VI) concentration was computed by fitting with an exponential function, and the fitted curves are shown in **(B,C)** as spline traces. Detailed parameters are tabulated in the text. In each panel, the left axis represent the OD and chromate concentrations are shown on right axis.

All three organisms were grown with nitrate (20 mM), as the terminal electron acceptor with either acetate (*G. metallireducens*) or lactate (*S. barnesii* and *D. desulfuricans*) as the electron donor and carbon source. Under these conditions all three organisms grew, with both *G. metallireducens* and *D. desulfuricans* reaching stationary phase later (4 and 2 days, respectively) than *S. barnesii* (1 day). From the optical density measurements (absorption at 600 nm), *D. desulfuricans* cultures yielded two times larger cell mass that the other two organisms (OD’s of ∼0.4 in *D. desulfuricans*, and 0.2 in *S. barnesii* and *G. metallireducens*), even though similar inoculum volumes was used in all cases (*S. barnesii, D. desulfuricans*, 3 mL of 48 h cultures, *G. metallireducens*, 3 mL of 5-day culture). Addition of chromate (100 μM) to nitrate enriched media influenced the growth of all three organisms but differently (Figure 1).

*G. metallireducens* growth on nitrate was completely inhibited by the presence of 100 μM Cr(VI) for the duration of the experiment (168 h, Figure 1A), and no increase in cell density was detected. Interestingly, approximately 40% of Cr(VI) was transformed over the 7 day of incubation (Figure 1A) despite the lack of bacterial growth. In contrast, control experiments with uninoculated FWA-NO_3_ medium showed no significant reduction of chromium.

*D. desulfuricans* growth was altered by the presence of Cr(VI). Cr(VI) decreased the growth rate of *D. desulfuricans* by half, but did not affect the final growth yields (Figure 1B). During the prolonged lag phase Cr(VI) was rapidly reduced within the first 24 h and was below detection levels after 10 days (Figure 1B). Initially, there was no concomitant growth of cells and decrease in Cr(VI) concentration, but the culture was well into exponential growth phase by 72 h. Thus it appeared that the concentration of Cr(VI) had to be reduced below 40 μM before a robust respiratory growth on nitrate could begin (Figure 1B). A similar Cr(VI) induced lag phase has been seen in *Desulfovibrio vulgaris* when grown on sulfate (Klonowska et al., 2008). While uninoculated SES3 medium can also reduce chromate (∼70%) over a period of 120 h, the biotic reduction (8.45 × 10^−2^ h^−1^) occurs at twice the abiotic rate (3.89 × 10^−2^ h^−1^; Table 1). Furthermore, when the experiments were repeated using FWL-NO_3_ medium, little abiotic Cr(VI) reduction was observed and the growth yields were comparable (Zhang, 2012).

**Table 1:** **Parameters obtained from non-linear curve fitting**.

Organism/condition	*R*^2^	Parameters			Rate/h
		*y*_0_	*A*_1_	*t*_1_	
*D. desulfuricans* in SES3 medium, live cells	0.948	8.06 (±3.68)	90.99 (±7.44)	11.84 (±2.55)	8.45 × 10^−2^
*D. desulfuricans* in SES3 medium, heat killed	0.995	4.18 (±0.92)	95.65(±2.64)	1.14 (±0.10)	87.72 × 10^−2^
*S. barnesii* in SES3 medium, live cells	0.952	2.85 (±8.51)	104.73 (±9.26)	29.09 (±8.10)	3.44 × 10^−2^
*S. barnesii* in SES3 medium, heat killed	0.971	10.49 (±4.68)	80.92 (±5.39)	25.20 (±5.56)	3.97 × 10^−2^
SES3 medium only. Cr(VI) measurement by DPC method	0.979	24.30 (±4.24)	74.70 (±6.52)	25.72 (±5.97)	3.89 × 10^−2^
SES3 medium only. Cr(VI) measurement by ICP-MS method	0.955	28.08 (±4.94)	69.27 (±8.81)	19.79 (±6.95)	5.05 × 10^−2^
SES3 medium. Cr(III) measurement by ICP-MS method	0.955	71.92 (±4.94)	−69.27 (±8.81)	−19.79 (±6.95)	5.05 × 10^−2^

*S. barnesii* exhibited little difference in growth kinetics or cell yield between cells grown on nitrate in the presence or absence of Cr(VI) (Figure 1C). The rate of Cr(VI) reduction also seemed to coincide with the increase in cell mass, with the Cr(VI) concentration leveling off at around 10 μM as the cells went into stationary phase, suggesting that *S. barnesii* has an efficient cellular mechanism available for Cr(VI) transformation. It is interesting to note that in a previous study, *S. barnesii* was shown to concomitantly reduce selenate (in μM concentrations) while growing on nitrate (in mM concentrations) as the organism expressed both selenate and nitrate reductase enzymes (Oremland et al., 1999). Although the rate of change in Cr(VI) concentration reduction was significant in the inoculated media, *S. barnesii* cells were able to remove Cr(VI) more efficiently [*S. barnesii* decreased Cr(VI) concentration below 10 μM within 48 h, while the uninoculated media required 120 h to reach 10 μM. Figure shows 20 μM].

### Chromate reduction in live cells, spent medium, and with heat killed cells

**Figure 2:**
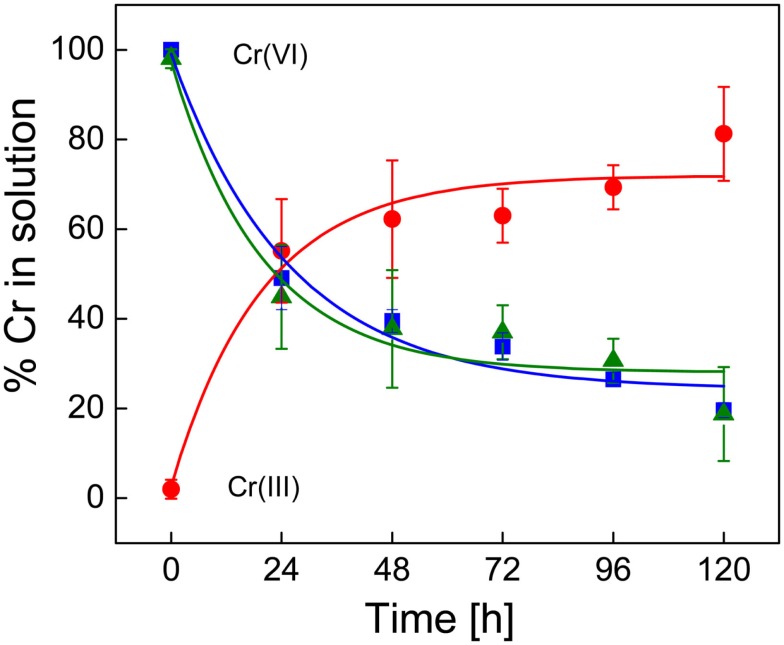
**Chromium(VI) reduction in uninoculated SES3 medium as determined by the diphenylcarbazide method (■) for Cr(VI) and by the isotope dilution method with ICP-MS for Cr(VI) (▲) and Cr(III) (●)**. Each of the measurements was done in triplicate with standard. The spline curves represent non-linear curve fitting.

Initially, the chromium concentration was determined using the traditional DPC assay which is specific for Cr(VI). However, we also used isotope dilution ICP-MS, which can measure both Cr(III) and Cr(VI) to confirm that Cr(III) was formed and to validate the colorimetric DPC assay. Concentrations of Cr(VI) in the uninoculated FWL-NO_3_ media were not significantly different among the ICP-MS and DPC methods. The ICP-MS measurements also demonstrated that Cr(VI) is stoichiometrically reduced to Cr(III) (Figure 2). The rate of increase (5.3 × 10^−2^ h^−1^) in Cr(III) concentration was also comparable with that of the decrease in Cr(VI) concentration. Furthermore, the ICP-MS data also indicate that under the experimental conditions, results from the DPC assay and ICP-MS are comparable, as both analyses provided a Cr(VI) reduction rate of 5.9 × 10^−2^ h^−1^ (assuming a first order process). Thus, kinetically, the data suggest that under the experimental conditions, Cr(VI) is reduced only to Cr(III), a process that requires three electrons. In all subsequent measurements the DPC method was used, and assumed that Cr(VI) was reduced to Cr(III) given the absence of precipitate in the spent culture medium.

**Figure 3:**
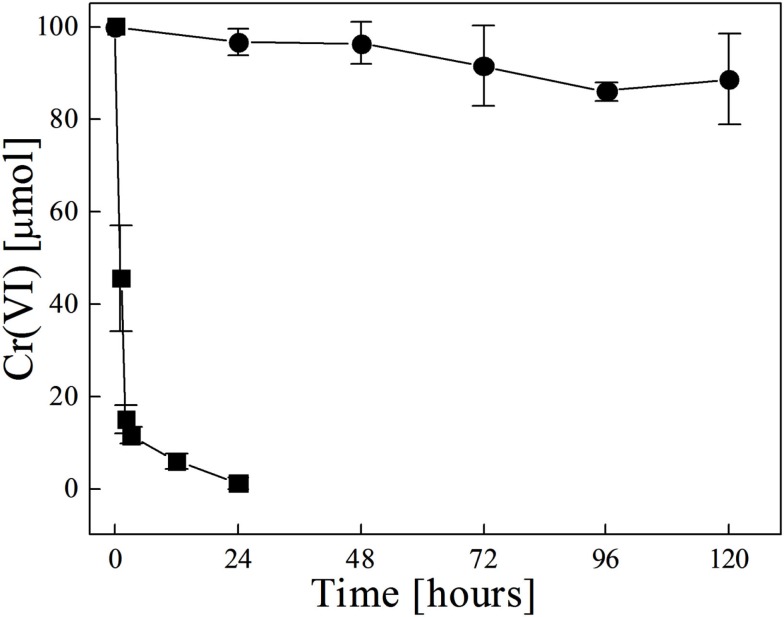
**Chromate reduction in cultures of *G. metallireducens* and the filtered spent medium**. The cells grown on nitrate for 60 h were exposed to 100 μM chromate then incubated for 120 h. Whole cells (■) and filtered spent medium (●).

Because *G. metallireducens* did not grow on nitrate media in the presence of Cr(VI) (Figure 1), the disappearance of Cr(VI) over time suggested the cells might still be able to reduce chromium. In order to investigate this possibility, cultures of *G. metallireducens* were grown on nitrate for 60 h, then were exposed to 100 μM Cr(VI), and incubated for an additional 120 h. Under these conditions, Cr(VI) was rapidly reduced by the cells to non-detectable levels by 24 h (Figure 3); however the cells could not continue to grow past 24 h. To confirm that the bacteria are required for Cr(VI) reduction, the medium was filtered to remove live cells from the 60 h cultures and then exposed to Cr(VI). The spent medium showed little Cr(VI) reduction (Figure 3). These results indicate that live cells of *G. metallireducens* are required for Cr(VI) reduction, and that extracellular metabolites are not involved.

**Figure 4:**
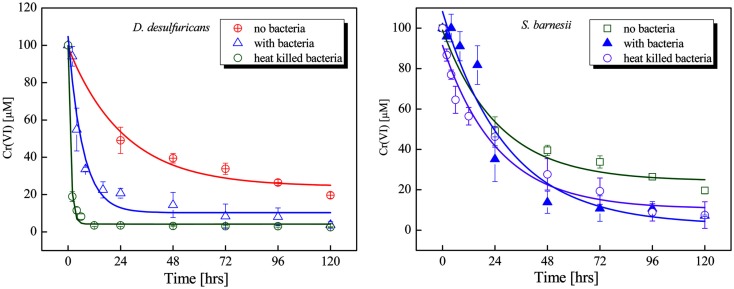
**Chromate reduction in cultures of *D. desulfuricans* (left panel) and *S. barnesii* (right panel) for live bacteria (▲) and heat killed bacteria (○) as compared to the uninoculated SES3 medium (⊕ or □)**. The spline curves are non-linear fit of the data from which the rate values were calculated.

Chromate reduction was also examined in heat killed cells. Cells of *D. desulfuricans* and *S. barnesii*, grown on SES3 medium were harvested after 48 h of growth, then autoclaved and transferred to fresh media with amended Cr(VI) to examine chromate reduction activity by heat killed cells (Figure 4). For comparison, Figure 4 also shows the chromate reduction with medium only and with live cells. For *S. barnesii* cells, the rate of chromate reduction with the live cells is marginally faster than that with the medium only. The rate of chromate reduction with heat killed cells is comparable to that obtained with live bacteria. In contrast, the heat killed *D. desulfuricans* cells exhibit a much more rapid reduction of Cr(VI) (Table 1).

**Figure 5:**
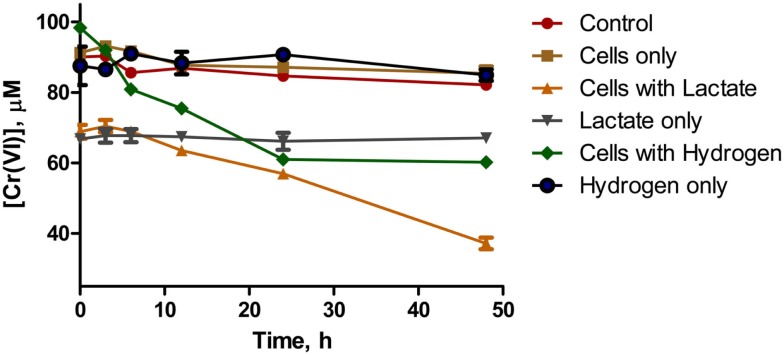
**Chromate reduction in whole cells of *D. desulfuricans* provided with lactate (▲) or hydrogen (♦) as electron donor**. Controls were Cr(VI) with no cells or donor (○), Cr(VI) with cells only (■), Cr(VI) with lactate only and no cells (▽), Cr(VI) with hydrogen only and no cells (●).

Whole cells of *D. desulfuricans* grown on nitrate showed little Cr(VI) reduction activity, even when provided a source of electron donor (e.g., lactate, H_2_; Figure 5). However, the filtered spent medium rapidly reduced Cr(VI) (Table 2). Caution was exercised as it was found that lactate alone (in the controls) reduced a certain amount (20–30%) of Cr(VI) in solution. Nevertheless, autoclaved cultures (that contained both heat killed cells and spent medium) and autoclaved filtered spent medium exhibited greater rates of Cr(VI) reduction indicating that this activity was enhanced by heating (Table 2). In addition, this activity was concentration dependent as demonstrated by serial dilution (Zhang, 2012). These results indicate that although viable cells do possess the ability to reduce Cr(VI), in culture, the main mechanism of resistance is through a heat stable secreted metabolite. Ongoing studies from our laboratories will further elucidate this point.

**Table 2:** **Chromate reduction activity in spent medium of nitrate grown cultures of *D. desulfuricans***.

Time(h)/Cr(VI) (μM)	HKC	HK-FSM	FSM
0	100	100	100
2	5.51	4.08	19.4
6	0	0	4.48
12	0	0	4.02
24	0	0	0
48	0	0	0

*The average from four independent experiments is shown. HKC – heat killed (autoclaved) culture, FSM – filtered spent medium, HK-FSM – heat killed (autoclaved) filtered spent medium*.

Both *S. barnesii* cells grown on nitrate and the filtered spent medium could reduce Cr(VI). Similar to the results seen for *D. desulfuricans* strain 27774, autoclaved cultures, and filtered spent medium alone showed this activity to be enhanced after heating (Table 3). Thus *S. barnesii* chromate reduction appears to be both cell associated and extracellular.

**Table 3:** **Chromate reduction activity in spent medium of nitrate grown cultures of *S. barnesii***.

Time(h)/Cr(VI) (μM)	HKC	HK-FSM	FSM
0	100	100	100
2	57.33	47.56	64.49
6	54.67	37.80	62.31
12	32.0	25.61	61.59
24	8.0	11.83	42.03
48	0	4.88	26.81

*The average from four independent experiments is shown. HKC – heat killed (autoclaved) culture, FSM – filtered spent medium, HK-FSM – heat killed (autoclaved) filtered spent medium*.

### Chromium reduction by protein fractions

**Figure 6:**
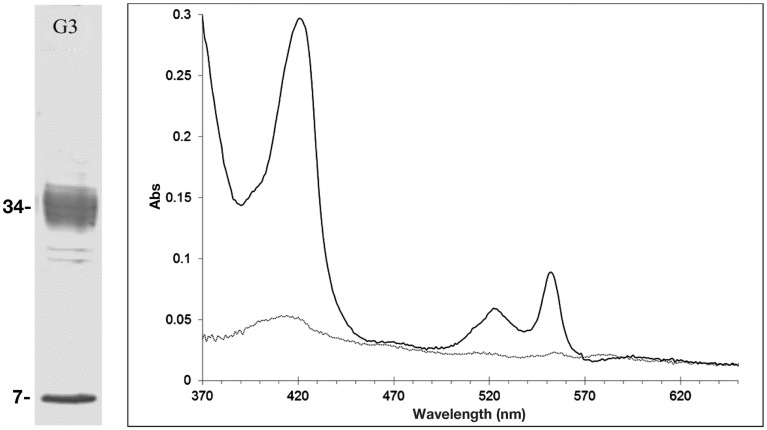
**The multiheme cytochrome c annotated as hypothetical protein Gmet_0913 from *G. metallireducens***. Left panel, SDS-PAGE (G3 refers to fraction G3 of the preparatory electrophoresis). Right panel, the UV/Vis electronic spectra of fraction G3, showing the as-isolated (grey line) and the dithionite reduced spectrum (black line).

To determine whether Cr(VI) was inhibiting growth though the inhibition of nitrate and/or nitrite reductase enzymes, we examined the nitrate and nitrite reductase activity with cell fractions and purified proteins. Both nitrate and nitrite reductase activities were affected by the presence of Cr(VI), with nitrite reductase activity inhibited to varying extents in all three organisms. Dissimilatory sulfite reductase (dSiR), which can reduce nitrite, was enriched from cells of *D. desulfuricans* grown on nitrate (Lui et al., 1994). We found that dSiR nitrite reduction was inhibited (∼40% reduction) by the presence of Cr (VI). No direct chromate reduction was detected in any protein fractions, suggesting that Cr(VI) reduction in *D. desulfuricans* is not enzyme catalyzed. The rate of nitrite reduction in cell lysates of *G. metallireducens* in the presence of Cr(VI) was least affected, with only a 10% decrease in activity as compared to nitrite alone. In the process of purifying components of the DNRA pathway from *G. metallireducens* grown on nitrate, a multiheme c-type cytochrome that exhibited nitrite reductase activity was isolated. A prominent pink band that separated in preparative gels was found to contain two proteins of relative molecular masses of 34 and 9 kDa respectively (Figure 6). The larger protein was identified by MALDI-TOF to be annotated as the hypothetical protein Gmet_0913 (accession number 78222133). The sequence was found to contain nine heme-c binding (CXXCH) motifs. The electronic spectrum was also consistent with a c-type cytochrome with absorbance maxima at 408, 522, and 551 nm for the dithionite reduced protein (Figure 6). In addition, the dithionite reduced cytochrome could be oxidized with nitrite or Cr(VI). Furthermore, nitrite reductase activity as measured using the methyl viologen assay indicated the activity was affected by chromate (0.7 mM). Future research with isolated enzymes to establish the significance of this enzyme in nitrite reduction is planned.

**Figure 7:**
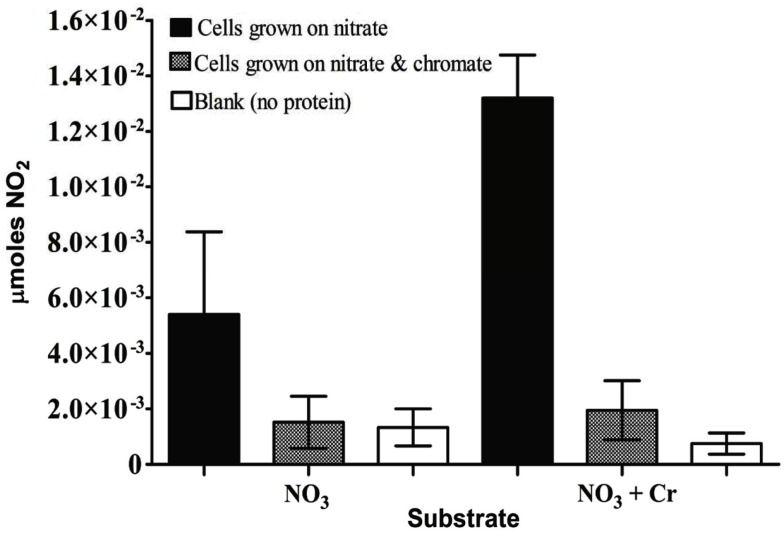
**The effect of chromate on nitrate reductase activity in *S. barnesii* cell lysates**. The activity was determined for lysates prepared from cells grown on nitrate medium alone (black bars) or amended with chromate (gray bars). The blank (white bars) contained the reaction mixture without protein.

**Figure 8:**
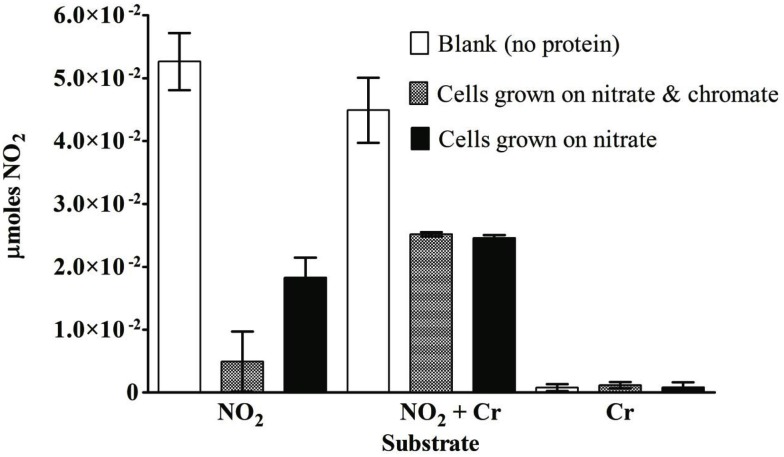
**The effect of chromate on the nitrite reductase activity in *S. barnesii* cell lysates**. The activity was determined for lysates prepared from cells grown on nitrate medium alone (black bars) or amended with chromate (gray bars). The blank (white bars) contained the reaction mixture without protein.

**Figure 9:**
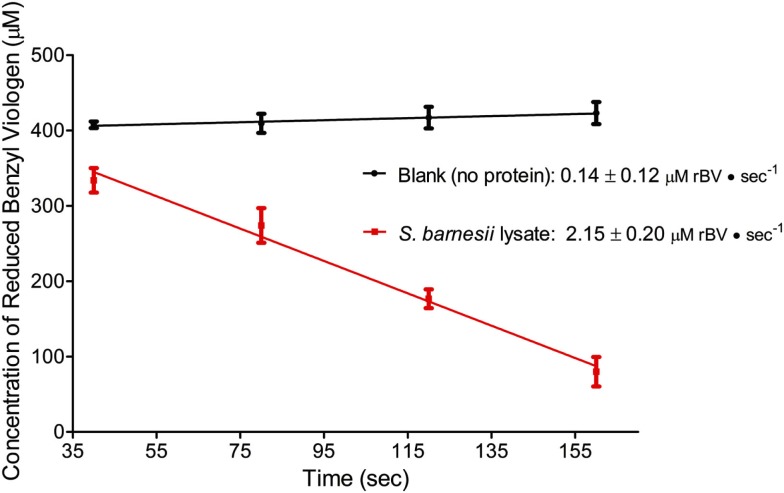
**The oxidation of reduced benzyl viologen by *S. barnesii* lysate in the presence of chromate**.

For *S. barnesii*, the effect of Cr(VI) on nitrate and nitrite reductase activity was dependent on whether the cells had been exposed to Cr(VI) during growth. Cell lysates from cells grown on nitrate and Cr(VI) exhibited less activity than the cells grown on nitrate alone. Interestingly, when chromate was added to the reaction mixture, nitrate reductase activity was higher in cells grown on nitrate alone while Cr(VI) had no effect on nitrate reductase activity in cells grown on nitrate and chromate (Figure 7). Conversely, nitrite reductase activity was greater (47%) in cell lysates of cultures grown on nitrate in the presence of Cr(VI) than those grown on nitrate alone (Figure 8). Nitrite reductase activity was inhibited, however, by the presence of Cr(VI) in the reaction mixture regardless of whether the cells had been grown in the presence or absence of Cr(VI). Lastly, cell lysates of *S. barnesii* could reduce Cr(VI) (Figure 9) as evidenced by the rapid (within 200 s) and complete oxidation of reduced benzyl viologen. These data suggest that chromate can be enzymatically reduced by *S. barnesii*.

**Figure 10:**
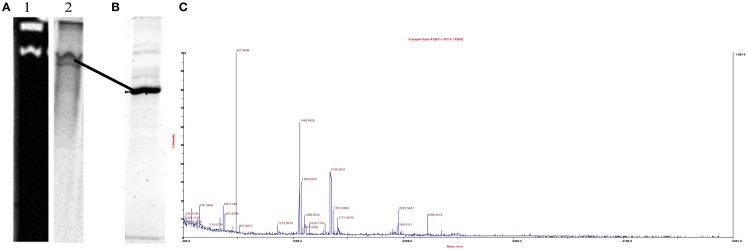
**Reductase activity and identification of the non-specific metalloid reductase Rar from *S. barnesii***. **(A)** Native gel of OBGP (1%) solubilized membrane proteins developed with nitrite showing two bands of activity (lane 1). Lane 2 is the same gel stained with Coomassie blue. The lower active (clear) band is Rar **(B)** SDS-PAGE of lower active band excised from **(A)** (shown by line) **(C)** MALDI-TOF MS identification the protein excised from gel in **(B)** as Rar.

In order to further elucidate the cellular mechanism for Cr(VI) reduction by *S. barnesii*, solubilized membrane proteins were subjected to both CN-PAGE activity assays and sequential ammonium sulfate precipitation followed by DEAE ion chromatography. In-gel activity assays indicated that two different protein bands could couple methyl viologen oxidation to nitrite reduction as these proteins were clearly separated on the native gel (Figure 10). A non-specific redox active protein, Rar, was subsequently identified by MALDI-TOF MS of the excised lower active band after purification to homogeneity in a second dimension SDS-PAGE (Figures 10B,C). Rar could be solubilized with the detergent OBGP and highly enriched fractions could be obtained with subsequent ammonium sulfate precipitation as it remained soluble at 75% saturation (Sparacino-Watkins, 2011). In addition to a nitrite and variety of metalloids (e.g., selenate, selenite, arsenate) as had been previously determined (Stolz and Oremland, 1999), in-gel assays indicated that Rar could also couple the oxidation of methyl viologen to the reduction of Cr(VI) (Sparacino-Watkins, 2011).

## Discussion

The growth experiments clearly showed that under nitrate respiring conditions, the three organisms responded differently. We had originally hypothesized that Cr(VI) reduction by *G. metallireducens* would not occur under dissimilatory nitrate reducing conditions, as nitrate reduction via Nar is more thermodynamically favorable than Cr(VI) reduction (Stolz et al., 1999; Ackerley et al., 2004). Instead, the cells could not grow in the presence of Cr(VI). Nevertheless, Cr(VI) was slowly reduced over time in these cultures. The results that whole cells, but not filtered spent medium, could reduce Cr(VI) suggests that *G. metallireducens* utilizes a cell associated mechanism for Cr(VI) reduction but cannot efficiently detoxify Cr(VI) for normal growth.

Previous investigations of nitrate respiration in *G. metallireducens* revealed that a low potential cytochrome, putatively identified as Nrf, could be oxidized by Cr(VI) (Lin, 1994; Martínez Murillo et al., 1999). This work here revealed an additional candidate c-type cytochrome, Gmet_0913, that not only could couple nitrite reduction to methyl viologen oxidation, but was also oxidized by Cr(VI). Thus, it is plausible that Cr(VI) can inhibit the DNRA pathway by oxidizing cytochromes, like Nrf. Our future studies will investigate the effect of Cr(VI) of nitrite reduction with the isolated enzymes, as it is not clear if cytochrome c oxidation by Cr(VI) produced Cr(III). A second plausible source of the Cr(VI) reduction in *G. metallireducens* may be chemotaxis and motility proteins. It is also possible that the conductive pili may be involved in Cr(VI) reduction. We have found that chemotaxis and motility proteins are up-regulated in chromate exposed cells (Basu et al., 2010; Chovanec et al., 2010). A similar mechanism has been reported for *Enterobacter cloacae* (Yang et al., 2007). An alternative mechanism is that Cr(VI) is reduced by Fe(II) produced through dissimilatory iron reduction. Cr(VI) reduction by Fe(II) is well established (Kamaludeen et al., 2003; Cheng et al., 2010; Xu et al., 2012). Amending the FWA-NO_3_ medium with ferric citrate did indeed result in the reduction of Cr(VI) however, whether the process is abiotic or biologically induced remains to be determined.

The growth experiments with *D. desulfuricans* showed that Cr(VI) initially inhibits growth on nitrate, but that growth can commence after a short lag phase. Once the Cr(VI) concentration fell below 40 μM, exponential growth began. Interestingly, whole washed cells showed little Cr(VI) reduction activity, whether an exogenous electron donor such as hydrogen or acetate was provided. Most of the activity was in the filtered spent medium. Investigation into possible enzymatic origins for this activity indicated that Cr(VI) does inhibit nitrite reduction by enriched protein fractions, such as the dSiR enzyme. Furthermore, that Cr(VI) could oxidize dSiR, suggests that Cr(VI) may act non-specifically by oxidizing redox active proteins, thus altering the redox poise and inducing oxidative stress, similar to what was observed in *G. metallireducens*. A search of the annotated genome of *D. desulfuricans* strain ATCC 27774 revealed that it does possess a homolog of [NiFe] hydrogenases that have been shown to reduce Cr(VI) in other organisms. In addition, our results using hydrogen as the electron donor (Figure 5) indicate that the amount of Cr(VI) reduced by hydrogenase is relatively small. Thus, the bulk of Cr(VI) reduction is not enzymatic, but rather appears to be mediated by a heat stable, redox active secondary metabolite secreted into the medium.

From the *S. barnesii* growth experiments we found that this organism can simultaneously reduce Cr(VI) while respiring nitrate. The enzyme assays indicate that *S. barnesii* cell lysate can reduce Cr(VI), as demonstrated by the viologen-coupled reductase assay. Also, it appears that the presence of chromate in the growth medium can actually increase nitrite reduction by *S. barnesii* cell lysates, compared to cells grown on nitrate alone. Still nitrite reduction is inhibited by the presence of Cr(VI) in the assay, independent of the growth conditions [with or without Cr(VI) in the media]. This result lead us to suggest that the living cells have a mechanism to detoxify Cr(VI), presumably protecting the sensitive redox active proteins like Nrf from oxidative stress, as lysing the cells makes the enzymes more sensitive to Cr(VI). Thus physiologically there must be a separate mechanism for Cr(VI) reduction such as Rar (*vide infra*).

In-gel activity assays revealed that Rar can couple methyl viologen oxidation to chromate reduction. While the Rar catalyzed chromate reduction was not present in the liquid protein assays, it is a potential source of the activity seen in whole washed cells. In addition, assays with filtered spent medium indicate that redox active secondary metabolites are also secreted into the medium. Thus the combination of the secreted metabolite and the presence of a non-specific metalloid reductase (Rar) may provide resistance to Cr(VI) allowing the components of nitrate respiration (e.g., Nap, Nrf) to continue to function.

## Conclusion

This investigation demonstrates that each organism, *G. metallireducens*, *D. desulfuricans*, and *S. barnesii*, reacts differently to chromate in the presence of nitrate. *G. metallireducens* and *D. desulfuricans* appear to co-metabolize Cr(VI) reduction while utilizing DNRA to fuel growth, yet each organism utilizes different molecular mechanism of chromate reduction. Our results suggest that Cr(VI) can influence the activity of the enzymes involved in DNRA, however, the exact nature is yet to be fully understood. Cytochromes, including the multiheme nitrite reductase (Nrf), can be readily oxidized by Cr(VI) thus rendering them inoperable. Providing an exogenous of source of reducing equivalents could help maintain cellular redox poise and enhance the rates of Cr(VI) reduction. Our results also demonstrated that each of these model organisms has a unique mechanism for Cr(VI) amelioration. In culture, *G. metallireducens* appears to reduce Cr(VI) either indirectly through the production of Fe(II) or by direct cell contact, perhaps through conductive pili. In nature, the elevated concentrations of iron associated with environments where *Geobacter* species are typically found allow for Cr(VI) reduction by biologically produced Fe(II). We suggest that *D. desulfuricans*, Cr(VI) reduction and resistance involves a heat stabile and readily soluble secreted metabolite; direct cell contact is not required. The identity of this compound and the nature of its reactivity toward Cr(VI) remain to be resolved. Lastly, *S. barnesii* is highly resistant to Cr(VI) as it possesses both a cell associated reductase and a redox active secreted metabolite. The ability to simultaneously reduce Cr(VI) and other metals and metalloids (e.g., selenium) while growing by nitrate respiration suggests this organism may be useful in remediation strategies involving natural attenuation. To this extent it is encouraging to note that *Sulfurospirillum* species have indeed been detected at Cr(VI) contaminated sites. Further studies on chromium metabolism in all three organisms will be greatly enhanced by proteomic investigations facilitated by the availability of genomic data, investigations currently underway.

## Conflict of Interest Statement

The authors declare that the research was conducted in the absence of any commercial or financial relationships that could be construed as a potential conflict of interest.
